# Genome-wide analysis of citrus TCP transcription factors and their responses to abiotic stresses

**DOI:** 10.1186/s12870-022-03709-3

**Published:** 2022-07-06

**Authors:** Dong-Hai Liu, Yin Luo, Han Han, Yong-Zhong Liu, Shariq Mahmood Alam, Hui-Xing Zhao, Yan-Ting Li

**Affiliations:** Key Laboratory of Horticultural Plant Biology (Ministry of Education)/College of Horticulture & Forestry Sciences, Huazhong Agricultural University, Wuhan, 430070 P.R. China

**Keywords:** TCP family, *Citrus sinensis*, Abiotic stress, Genome-wide analysis, Expression pattern

## Abstract

**Background:**

Citrus is one of the most important fruit crops in the world, and it is worthy to conduct more research on artificially controlling citrus plant growth and development to adapt to different cultivation patterns and environmental conditions. The plant-specific TEOSINTE BRANCHED1, CYCOLOIDEA, and PROLIFERATING CELL FACTORS (TCP) transcription factors are crucial regulators controlling plant growth and development, as well as responding to abiotic stresses. However, the information about citrus TCP transcription factors remains unclear.

**Results:**

In this study, twenty putative *TCP* genes (*CsTCPs*) with the TCP domain were explored from *Citrus sinensis* genome, of which eleven (*CsTCP3*, − *4*, − *5*, − *6*, − *10*, − *11*, − *15*, − *16*, − *18*, − *19*, − *20*), five (*CsTCP1*, − *2*, − *7*, − *9*, − *13*), and four genes (*CsTCP8*, − *12*, − *14*, − *17*) were unevenly distributed on chromosomes and divided into three subclades. *Cis*-acting element analysis indicated that most *CsTCPs* contained many phytohormone- and environment-responsive elements in promoter regions. All of *CsTCPs* were predominantly expressed in vegetative tissues or organs (stem, leaf, thorn, and bud) instead of reproductive tissues or organs (flower, fruit, and seed). Combined with collinearity analysis, *CsTCP3*, *CsTCP9*, and *CsTCP13* may take part in leaf development; *CsTCP12* and *CsTCP14* may function in shoot branching, leaf development, or thorn development; *CsTCP15* may participate in the development of stem, leaf, or thorn. In mature leaf, transcript levels of two *CsTCPs* (*CsTCP19*, − *20*) were significantly increased while transcript levels of eight *CsTCPs* (*CsTCP2*, − *5*, − *6*, − *7*, − *8*, − *9*, − *10*, − *13*) were significantly decreased by shading; except for two *CsTCPs* (*CsTCP11*, − *19*), *CsTCPs*’ transcript levels were significantly influenced by low temperature; moreover, transcript levels of two *CsTCPs* (*CsTCP11*, − *12*) were significantly increased while five *CsTCPs*’ (*CsTCP14*, − *16*, − *18*, − *19*, − *20*) transcript levels were significantly reduced by drought.

**Conclusions:**

This study provides significant clues for research on roles of *CsTCPs* in regulating citrus plant growth and development, as well as responding to abiotic stresses.

**Supplementary Information:**

The online version contains supplementary material available at 10.1186/s12870-022-03709-3.

## Background

Transcription factors are proteins that play a pivotal role in plant growth and development by binding to promoter or enhancer regions of specific genes [1]. TCP family, a plant-specific transcription factor family, is originally named from the first four family members, TEOSINTE BRANCHED1 (TB1) in *Zea mays*, CYCOLOIDEA (CYC) in *Antirrhinum majus*, PROLIFERATING CELL FACTORS 1 and 2 (PCF1 and PCF2) in *Oryza sativa*, which contains a highly conserved non-canonical basic helix-loop-helix motif designated as the TCP domain with about 59 amino acids at the N-terminus; TCP domain is involved in nuclear targeting, DNA binding, and pair-wise protein-protein interaction [2].

TCP family exists widely in plants. There are five to six members in pluricellular green algae, mosses, ferns, and lycophytes [3, 4], and over ten members in angiosperms [2]. They are generally divided into two subfamilies, CLASS I (PCF or TCP-P subclass) specifically binding to GGNCCCAC, and CLASS II (TCP-C subclass) specifically binding to G(T/C)GGNCCC, based on the homology and variation of TCP domain [5]. Moreover, CLASS II subfamily can be further divided into CINCINNATA (CIN) and CYC/TB1 subclades [6]; several TCP members in CLASS II subfamily contain another conserved region named as the R domain, which is an arginine-rich motif and includes polar residues with hypothetical functions related to protein-protein interaction by forming a coiled coil [7].

As plant-specific transcription factors, TCP members, especially CLASS II subfamily members, play a crucial role in plant growth and development, such as plant height regulation [8, 9], lateral bud outgrowth [10, 11], thorn conversion [12], leaf morphogenesis [13, 14], trichome formation [15, 16], floral asymmetry [17, 18], pollen development [19, 20], embryo growth [21], seed germination [22, 23], and circadian rhythm [24]. On the other hand, TCP members can be regulated by endogenous signals such as phytohormones [25], and can also respond to exogenous factors such as abiotic stresses [26]. Moreover, previous studies indicated that some CIN subclade members can be mediated by *miR319* [27, 28].

As one of the most important fruit crops in the world, citrus produces fruits supplying not only different and vital nutrition for human health, but also tremendously delicious flavor for consumers [29]. With the update of labor-saving cultivation pattern, it is necessary to conduct more research on artificially controlling citrus plant growth and development. As mentioned above, some TCP members play a key role in regulation of plant height, lateral bud outgrowth, and leaf development. However, the information about citrus *TCP* genes is scarce, although complete genome sequence data of many citrus cultivars are released [30, 31]. In this study, a total of twenty putative *TCP* genes were explored from *Citrus sinensis* genome. The basic characteristics, gene duplication, phylogenetic relationships, *cis*-acting elements, gene ontology (GO) annotations, and protein-protein interaction were systematically analyzed for such *TCP* genes. In addition, their expression patterns were investigated in different tissues or organs as well as samples treated with shading, low temperature, and drought. Specially, *CsTCP3*, *CsTCP9*, and *CsTCP13* may function in leaf development; *CsTCP12* and *CsTCP14* may participate in the regulation of shoot branching, leaf development, or thorn development, respectively; *CsTCP15* may act as a regulator in the development of stem, leaf, or thorn; they possibly take part in the response of leaf to shading, low temperature, or drought, respectively. Overall, the present results suggested possible roles of some *TCP* genes and provided background information for further research on the specific role and mechanism of each *TCP* gene in citrus plant growth and development, as well as in response to abiotic stresses.

## Results

### Identification and basic characteristics of *Citrus sinensis TCP* genes

A total of twenty putative *TCP* genes were screened out from *Citrus sinensis* genome and named as *CsTCP1-20* according to their distribution order on chromosomes (Table 1). It was found that *CsTCPs* were unevenly distributed on chromosomes. In detail, chromosome 7 contained five *CsTCPs*, chromosomes 2 and 5 contained three *CsTCPs*, chromosomes 6, 8, and 9 contained two *CsTCPs*, chromosome 3 contained one *CsTCP*, and chromosomes 1 and 4 contained no *CsTCP*. However, the location of *CsTCP19* and *CsTCP20* on chromosomes was unclear. On the other hand, the number of amino acids (aa), molecular weight (Mw), and theoretical isoelectric point (pI) varied among CsTCPs (Table 1). The number of amino acids of CsTCPs was between 174 and 577. In detail, five CsTCPs contained less than 300 aa, thirteen CsTCPs contained 300 to 500 aa, and two CsTCPs contained more than 500 aa. Accordingly, their Mw varied largely either, from 19,507.20 Da (Da) to 61,307.81 Da. As for theoretical pI, it was between 4.44 and 9.47. The theoretical pI of eight CsTCPs was lower than 7, seven CsTCPs were from 7 to 9, and five CsTCPs were higher than 9. Moreover, no signal peptide was found in all CsTCP protein sequences, and the predicted subcellular localization was in nucleus (Table 1).

**Table 1:** Basic characteristics of *Citrus sinensis TCP* genes

Gene name	Gene ID	Chromosome location	Strand	Length of CDS (bp)	Number of amino acids	Molecular weight (Da)	Theoretical pI	Signal peptide length (aa)	Predicted subcellular localization
*CsTCP1*	Cs2g08080.1	chr2: 4848829..4853255	–	1515	504	55,291.28	7.16	–	Nuclear
*CsTCP2*	Cs2g15820.1	chr2: 12548474..12550882	+	1005	334	36,343.85	6.53	–	Nuclear
*CsTCP3*	Cs2g25640.1	chr2: 24866364..24868750	+	954	317	33,912.73	9.01	–	Nuclear
*CsTCP4*	Cs3g22260.1	chr3: 24926039..24927594	–	963	320	33,181.31	7.79	–	Nuclear
*CsTCP5*	Cs5g03980.1	chr5: 2195508..2197485	+	1281	426	46,428.83	7.10	–	Nuclear
*CsTCP6*	Cs5g10130.1	chr5: 6994168..6996231	–	1107	368	39,316.14	6.72	–	Nuclear
*CsTCP7*	Cs5g12070.1	chr5: 9052963..9054997	+	1098	365	40,856.09	6.74	–	Nuclear
*CsTCP8*	Cs6g18940.1	chr6: 18950739..18952107	+	897	298	34,061.36	9.38	–	Nuclear
*CsTCP9*	Cs6g22270.1	chr6: 21078708..21080763	+	1203	400	43,616.64	8.88	–	Nuclear
*CsTCP10*	Cs7g03980.1	chr7: 1887520..1889541	+	1185	394	42,105.60	7.78	–	Nuclear
*CsTCP11*	Cs7g11120.1	chr7: 7320850..7323133	+	825	274	29,286.77	9.44	–	Nuclear
*CsTCP12*	Cs7g12770.1	chr7: 8725990..8727399	–	1410	469	52,553.18	6.62	–	Nuclear
*CsTCP13*	Cs7g25460.1	chr7: 25950030..25952883	+	1281	426	46,861.16	6.78	–	Nuclear
*CsTCP14*	Cs7g26250.1	chr7: 26808458..26809706	–	1095	364	41,735.96	6.29	–	Nuclear
*CsTCP15*	Cs8g16060.1	chr8: 19154530..19155054	+	525	174	19,507.20	9.03	–	Nuclear
*CsTCP16*	Cs8g16080.1	chr8: 19169920..19171017	+	675	224	24,176.45	4.44	–	Nuclear
*CsTCP17*	Cs9g12640.1	chr9: 11059738..11061409	+	957	318	36,575.30	9.47	–	Nuclear
*CsTCP18*	Cs9g16600.1	chr9: 16080000..16081746	–	1017	338	35,966.34	8.84	–	Nuclear
*CsTCP19*	orange1.1 t02428.1	chrUn: 36970387..36971007	+	621	206	21,666.35	7.00	–	Nuclear
*CsTCP20*	orange1.1 t03896.1	chrUn: 59999964..60002615	+	1734	577	61,307.81	6.70	–	Nuclear

*aa* amino acid(s), *CDS* Coding sequence(s), *Da* Dalton(s), *pI* Isoelectric point

**Fig. 1:**
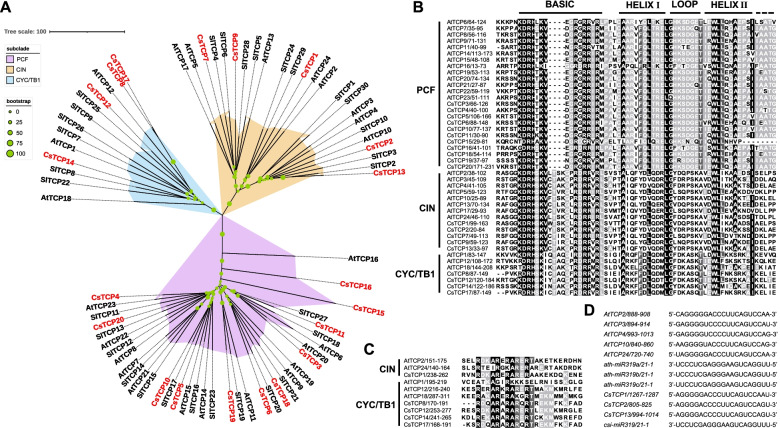
Phylogenetic analysis, conserved domain identification, and *miR319*-binding site recognition of *CsTCPs*. **A** Phylogenetic tree of *Citrus sinensis*, *Arabidopsis thaliana*, and *Solanum lycopersicum* TCP proteins. A total of seventy-four TCP protein sequences were analyzed by the Muscle method, and the neighbor-joining tree was constructed with MEGA X and iTOL v.6. Purple, orange, and blue regions represent PCF subclade, CIN subclade, and CYC/TB1 subclade, respectively. The putative CsTCPs are shown in red. The bootstrap test was implemented with 1000 iterations. Size of green dot represents bootstrap value. **B** Multiple sequence alignment of the TCP domain in *Citrus sinensis* and *Arabidopsis thaliana* TCP members. The alignment was performed by the Muscle method with MEGA X and was visualized using Jalview v.2.11.1.4. The conserved regions (BASIC, HELIX I, LOOP, and HELIX II) of TCP domain are indicated at the top. The conserved amino acids are in black. **C** Multiple sequence alignment of the R domain in TCP members. The conserved amino acids are in black. **D** Analysis of the putative target sites for *miR319* in *CsTCPs* and *AtTCPs*. The alignment was conducted using psRNATarget

In addition, these twenty TCP members could be divided into three subclades based on the homology and variation of their protein sequences by aligning with the protein sequences of *Arabidopsis thaliana* TCP (AtTCP) and *Solanum lycopersicum* TCP (SlTCP) transcription factors; eleven (CsTCP3, − 4, − 5, − 6, − 10, − 11, − 15, − 16, − 18, − 19, − 20), five (CsTCP1, − 2, − 7, − 9, − 13), and four members (CsTCP8, − 12, − 14, − 17) belonged to subclades PCF, CIN, and CYC/TB1, respectively (Fig. 1A). All the sequences contained the TCP domain by further aligning with AtTCP protein sequences (Fig. 1B). In BASIC region, Asp (D), His (H), Lys (K), and Arg (R) amino acid residues were completely conserved in the sequences of all members; the sequence of each PCF subclade member possessed a four-amino-acid deletion. In HELIX I and HELIX II regions, Leu (L) and Trp (W), two hydrophobic amino acid residues were fully conserved. In LOOP region, Gly (G), a hydrophilic amino acid residue was highly conserved. Moreover, the R domain with relatively conserved Ala (A) and Arg (R) amino acid residues was found in the sequences of one CIN subclade member (CsTCP1) and all CYC/TB1 subclade members (Fig. 1C); the putative *miR319*-binding sites were only found in the sequences of three *CIN*-type genes (*CsTCP1*, − *2*, − *13*) (Fig. 1D).

### Sequence features and protein-protein interaction of *Citrus sinensis* TCP members

**Fig. 2:**
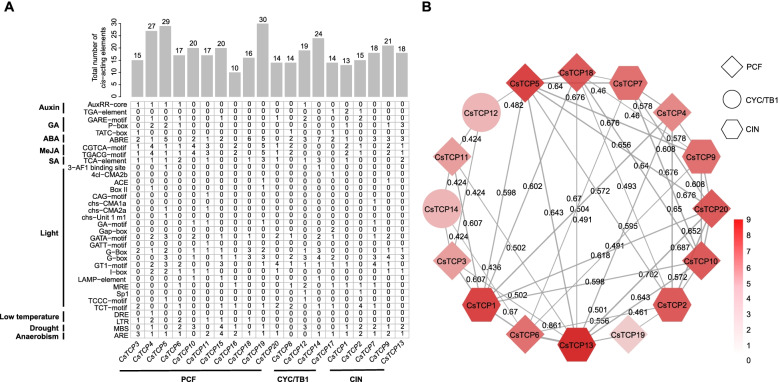
*cis*-acting elements and protein-protein interaction of *Citrus sinensis* TCP members. **A** Analysis of *cis*-acting elements in promoter regions of *CsTCPs*. The 2 kb upstream of translation initiation sites of twenty *TCP* genes were submitted to PlantCARE to screen out *cis*-acting elements. GA, gibberellin; ABA, abscisic acid; MeJA, methyl jasmonate; SA, salicylic acid. Gray bars represent the total number of *cis*-acting elements. Different numbers in grids represent the number of corresponding *cis*-acting elements. **B** Analysis of interaction among CsTCPs. All the sequences of twenty TCP proteins were submitted to STRING v.11.5 to analyze their interaction relationships, and the interaction network was constructed by Cytoscape v.3.6.1. Diamond, ellipse, and hexagon represent PCF subclade, CYC/TB1 subclade, and CIN subclade, respectively. Different color blocks represent the number of interaction proteins. Numbers in the network indicate the combined scores of two interaction proteins, and higher score corresponds to thicker line

Some conserved motifs were found in each CsTCP protein sequence, and their type or quantity varied greatly among subclades or members; gene structure, namely, the component and size of exon and intron was also different from each other (Fig. S1). Notably, thirty-four special *cis*-acting elements related to phytohormones and environmental signals were found in 2 kb promoter regions of twenty *CsTCPs*. However, each gene owned the different profile of *cis*-acting elements, and the number of *cis*-acting elements in the promoter region of each gene ranged from ten to thirty (Fig. 2A). Specially, *CsTCP19* contained 30 *cis*-acting elements, including ten methyl jasmonate (MeJA)-responsive elements, ten light-responsive elements, five abscisic acid (ABA)-responsive elements, three salicylic acid (SA)-responsive elements, one drought-responsive element, and one anaerobism-responsive element; whereas *CsTCP16* contained ten *cis*-acting elements, including four MeJA-responsive elements, three light-responsive elements, two anaerobism-responsive elements, and one drought-responsive element.

In addition, the interaction relationships among twenty CsTCPs were complex and sixteen members interacted with each other (Fig. 2B). Of them, CsTCP13 interacted with nine members; CsTCP1 and CsTCP5 interacted with eight members; CsTCP2, CsTCP10, CsTCP18, and CsTCP20 interacted with seven members; CsTCP6, CsTCP7, and CsTCP9 interacted with six members; CsTCP4 interacted with five members; CsTCP3 and CsTCP11 interacted with four members; CsTCP12 and CsTCP14 interacted with three members; CsTCP19 interacted with two members. Moreover, the highest interaction score (0.861) was found between CsTCP6 and CsTCP13.

### Collinearity analysis and GO annotation of *Citrus sinensis TCP* genes

**Fig. 3:**
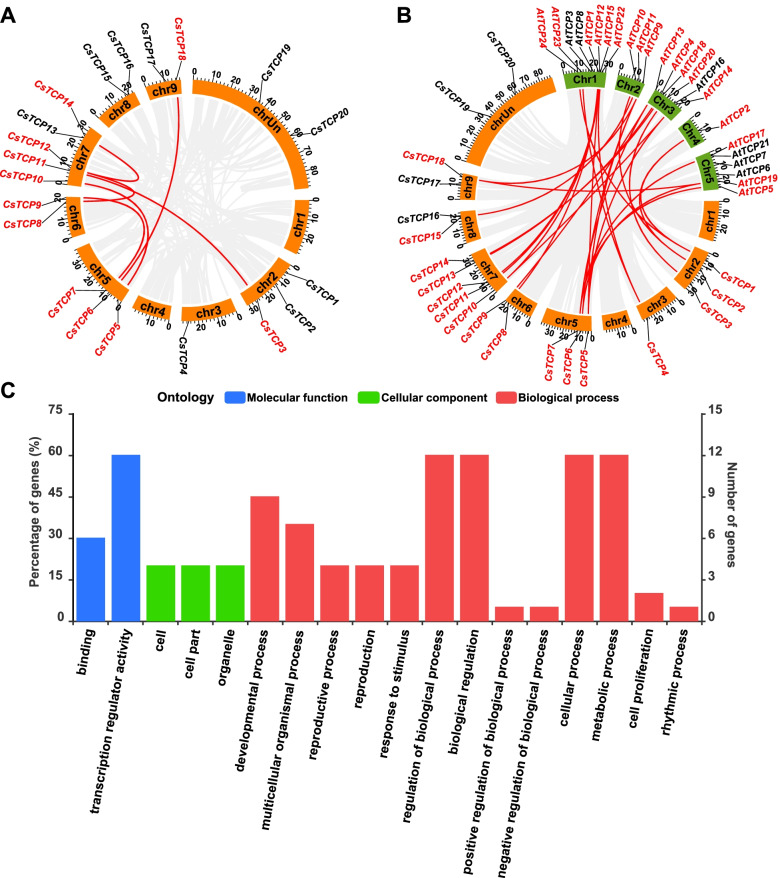
Collinearity analysis and gene ontology (GO) annotation of *CsTCPs*. **A** Paralogous relationships between *CsTCPs*. The twenty *TCP* genes were mapped on the chromosomes. Paralogs are shown in red and connected with red lines. Orange bands represent *Citrus sinensis* chromosomes. Tick labels represent chromosome length (Mb). **B** Orthologous relationships between *CsTCPs* and *AtTCPs*. The forty-four *TCP* genes were mapped on the chromosomes. Orthologs between two species are shown in red and connected with red lines. Orange and green bands represent *Citrus sinensis* and *Arabidopsis thaliana* chromosomes, respectively. Tick labels represent chromosome length (Mb). **C** GO annotations of *CsTCPs*. The sequences of twenty TCP proteins were submitted to EGGNOG-Mapper to perform GO annotation which was visualized by WEGO v.2.0. Blue, green, and red bars represent GO terms of molecular function, cellular component, and biological process, respectively

A total of eleven *CsTCPs* belonging to paralogs were distributed on chromosomes 2, 5, 6, 7, and 9, respectively (Fig. 3A). Of them, *CsTCP3* and *CsTCP11, CsTCP5* and *CsTCP10, CsTCP6* and *CsTCP18, CsTCP7* and *CsTCP9, CsTCP8* and *CsTCP12,* as well as *CsTCP12* and *CsTCP14* were paralogous genes, respectively. Moreover, the *K*a (nonsynonymous substitution rate)/*K*s (synonymous substitution rate) ratio of each pair was lower than 0.3 (Table S1). On the other hand, large-scale orthologous *TCP* genes including sixteen *CsTCPs* and eighteen *AtTCPs* were found between *Citrus sinensis* and *Arabidopsis thaliana* genomes (Fig. 3B). Specially, a sole orthologous relationship could be found in eight pairs of *TCP* genes, including *CsTCP3* and *AtTCP20*, *CsTCP8* and *AtTCP1*, *CsTCP9* and *AtTCP13*, *CsTCP11* and *AtTCP15*, *CsTCP12* and *AtTCP12*, *CsTCP13* and *AtTCP4*, *CsTCP14* and *AtTCP18*, as well as *CsTCP15* and *AtTCP11*.

In addition, a total of twelve *CsTCPs* (*CsTCP1*, − *2*, − *3*, − *4*, − *5*, − *8*, − *13*, − *14*, − *17*, − *18*, − *19*, − *20*) were annotated (Table S2). They could be classified into three classes, including molecular function, cellular component, and biological process (Fig. 3C). Of them, twelve *CsTCPs* were involved in transcription regulator activity, regulation of biological process, biological regulation, cellular process, and metabolic process; nine *CsTCPs* (*CsTCP1*, − *2*, − *3*, − *4*, − *5*, − *8*, − *13*, − *14*, − *17*) were related to developmental process; four *CsTCPs* (*CsTCP1*, − *5*, − *13*, − *18*) were involved in response to stimulus; two *CsTCPs* (*CsTCP1*, − *5*) were related to cell proliferation; one *CsTCP* (*CsTCP19*) was involved in rhythmic process.

### Spatio-temporal expression analysis of *Citrus sinensis TCP* genes

**Fig. 4:**
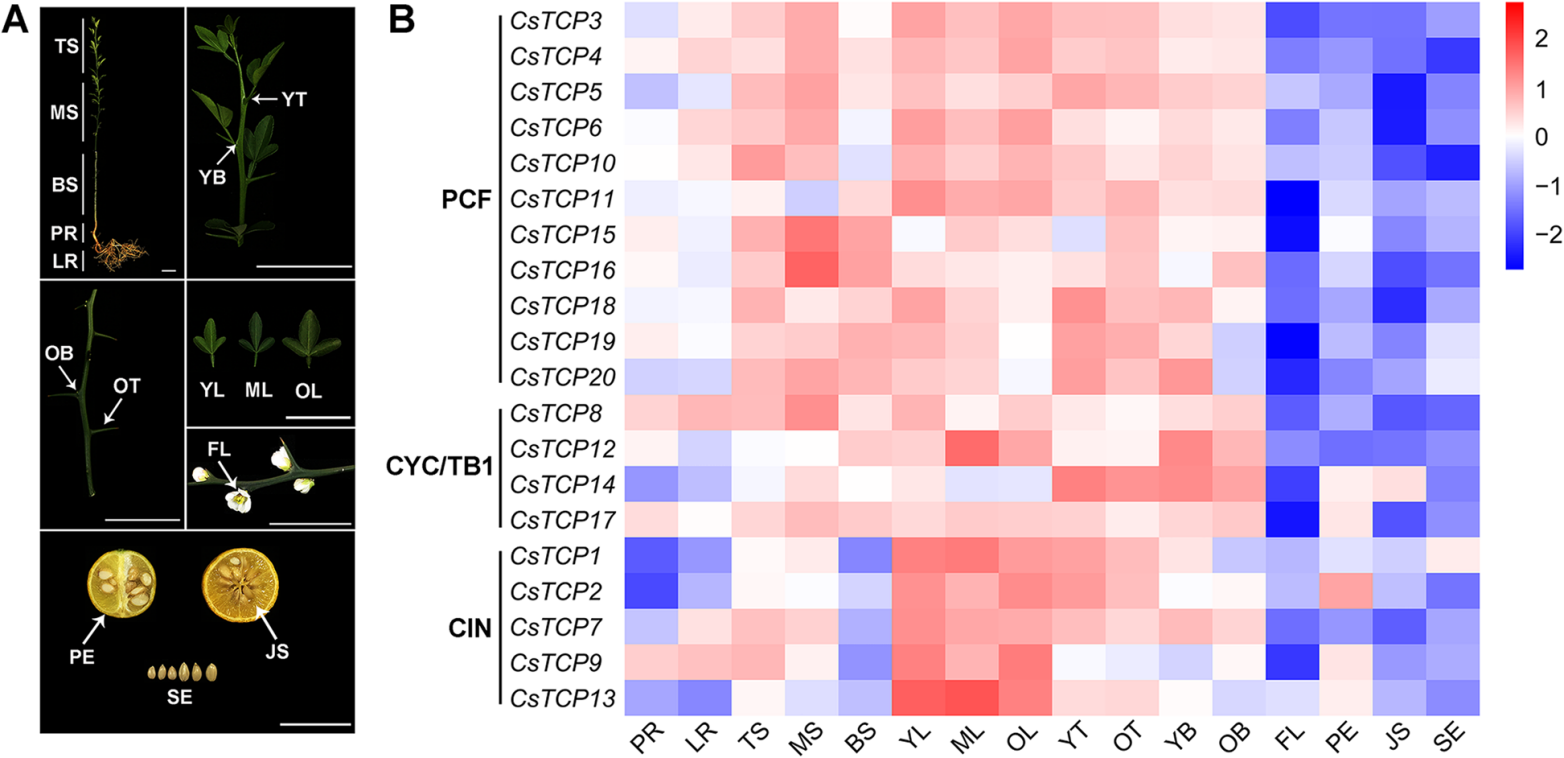
Expression profiles of *CsTCPs* in different tissues or organs. **A** Illustration of tissues or organs from *Poncirus trifoliata*. PR, primary root; LR, lateral root; TS, tip stem; MS, middle stem; BS, base stem; YL, young leaf; ML, mature leaf; OL, old leaf; YT, young thorn; OT, old thorn; YB, young bud; OB, old bud; FL, flower; PE, peel; JS, juice sac; SE, seed. Scale bars represent 5 cm. **B** Heat map of expression patterns. The results were the mean of three independent biological replicates with quantitative real-time PCR (qRT-PCR) technology and transformed by log2 fold change. Color scale represents relative expression level. Red represents high expression level, and blue represents low expression level

In different tissues or organs of *Poncirus trifoliata*, including root, stem, leaf, thorn, bud, flower, peel, juice sac, and seed (Fig. 4A), twenty *CsTCPs* exhibited multifarious spatio-temporal expression profiles. Most genes were highly expressed in stem, leaf, thorn, and bud, but were lowly expressed in root, flower, peel, juice sac, and seed (Fig. 4B). In detail, ten *CsTCPs* (*CsTCP1*, − *2*, − *3*, − *4*, − *6*, − *7*, − *9*, − *11*, − *12*, − *13*), six *CsTCPs* (*CsTCP5*, − *8*, − *10*, − *15*, − *16*, − *17*), three *CsTCPs* (*CsTCP14*, − *18*, − *19*), and one *CsTCP* (*CsTCP20*) showed the highest expression level in leaf, stem, thorn, and bud, respectively. On the other hand, eight *CsTCPs* (*CsTCP3*, − *9*, − *11*, − *14*, − *15*, − *17*, − *19*, − *20*), six *CsTCPs* (*CsTCP5*, − *6*, − *7*, − *8*, − *16*, − *18*), three *CsTCPs* (*CsTCP1*, − *2*, − *13*), two *CsTCPs* (*CsTCP4*, − *10*), and one *CsTCP* (*CsTCP12*) were expressed with the lowest level in flower, juice sac, root, seed, and peel, respectively. Moreover, *CsTCPs* from the same subclade also exhibited different expression profiles (Fig. 4B). For example, in CIN subclade, the expression level of *CsTCP1* in seed was similar to that in middle stem, while expression levels of the other genes in seed were lower than those in middle stem; on the other hand, expression levels of *CsTCP2* and *CsTCP9* were relatively high in peel and root, respectively, whereas expression levels of the other genes were relatively low in these two tissues or organs.

In addition, with the development of some tissues or organs, some genes presented different expression trends (Fig. 4B). Expression levels of five *CsTCPs* (*CsTCP3*, − *5*, − *8*, − *15*, − *16*) were increased from tip stem to middle stem, and then were decreased to base stem; however, expression levels of three *CsTCPs* (*CsTCP7*, − *9*, − *10*) were gradually decreased from tip stem to base stem. On the other hand, three *CsTCPs*’ (*CsTCP12*, − *13*, − *15*) transcript levels were increased from young leaf to mature leaf, and then were decreased to old leaf; while one *CsTCP*’s (*CsTCP18*) transcript level was decreased along with leaf development. As for in thorn, expression levels of five *CsTCPs* (*CsTCP7*, − *10*, − *14*, − *18*, − *20*) in young thorn were higher than those in old thorn, whereas the change in the expression level of *CsTCP15* was contrary. Similarly, expression levels of seven *CsTCPs* (*CsTCP7*, − *12*, − *13*, − *14*, − *18*, − *19*, − *20*) in young bud were higher than those in old bud, while the change of two *CsTCPs*’ (*CsTCP9*, − *16*) expression levels were contrary.

### Expression patterns of *Citrus sinensis TCP* genes responding to shading, low temperature, and drought

**Fig. 5:**
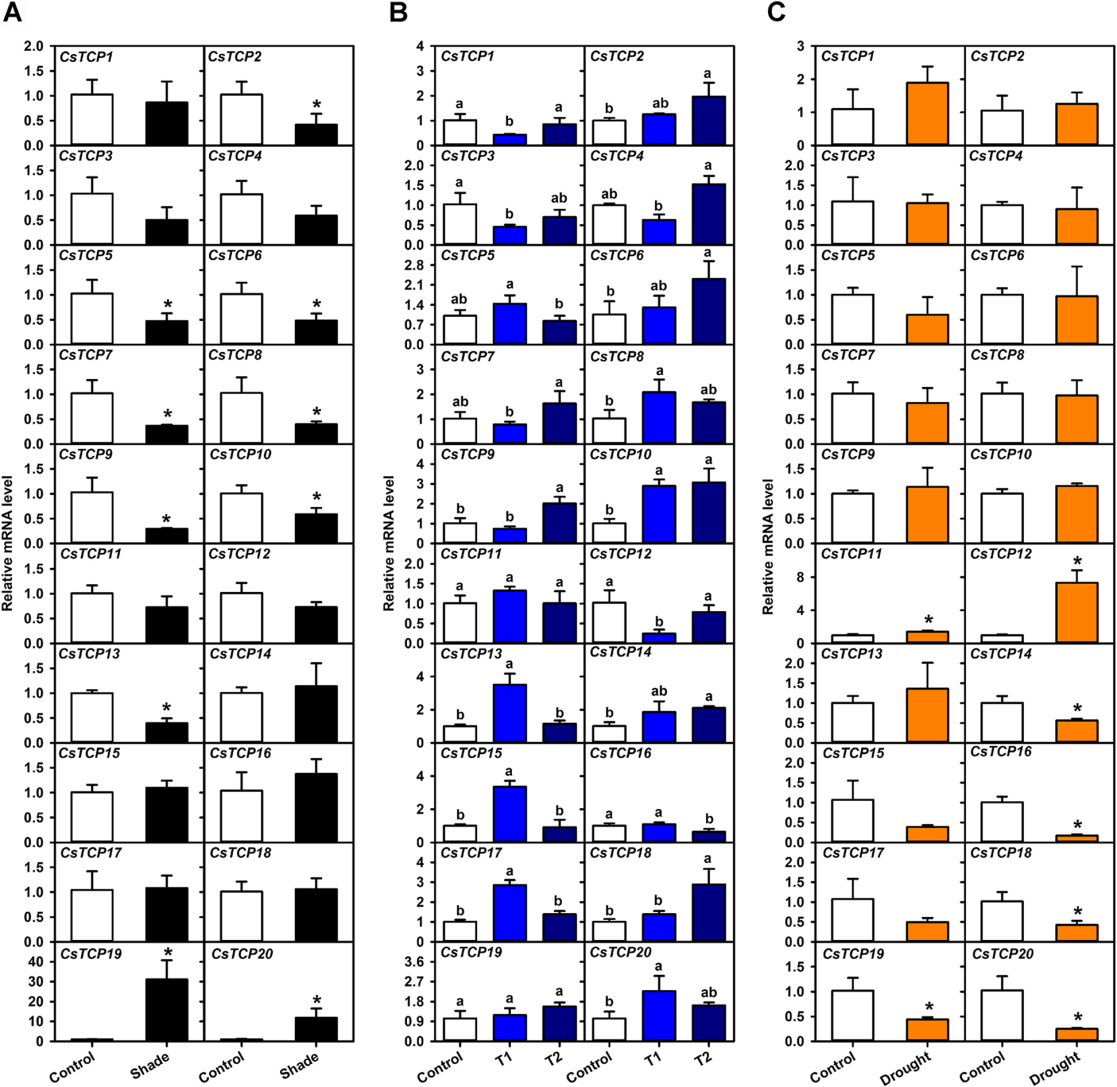
Expression patterns of *CsTCPs* in mature leaf responding to abiotic stresses. **A** Expression changes of *CsTCPs* under shade treatment. Non-shading was used as the control. **B** Expression changes of *CsTCPs* under low temperature treatments. Control represents the treatment for zero hour at 5 °C, T1 represents the treatment for 2 h at 5 °C, and T2 represents the treatment for 6 h at 5 °C. **C** Expression changes of *CsTCPs* under drought treatment. Results were the mean of three independent biological replicates with quantitative real-time PCR (qRT-PCR) technology. Error bars represent the standard deviation of replicates. The asterisk indicates statistically significant difference between groups at *P* < 0.05 by *t*-test. Different lower case letters indicate statistically significant difference among groups at *P* < 0.05 by Tukey test in ANOVA program

Shading significantly decreased the light intensity in the canopy (Fig. S2A), and influenced expression levels of ten *CsTCPs* in mature leaf of *Citrus reticulata* cv. Kinokuni (Fig. 5A). Of them, expression levels of two *CsTCPs* (*CsTCP19*, − *20*) were significantly increased and were about 31.0- and 11.7-fold higher than those in the control, respectively; on the contrary, expression levels of eight *CsTCPs* (*CsTCP2*, − *5*, − *6*, − *7*, − *8*, − *9*, − *10*, − *13*) were significantly decreased and were about 0.4, 0.5, 0.5, 0.4, 0.4, 0.3, 0.6, and 0.4 times of those in the control, respectively. On the other hand, expression levels of four *CsTCPs* (*CsTCP3*, − *4*, − *11*, − *12*) were down-regulated without significance (Fig. 5A).

Moreover, transcript levels of most *CsTCPs* could be obviously influenced by low temperature (Fig. 5B). Specifically, transcript levels of six *CsTCPs* were reduced at 2 h after 5 °C treatment and then were increased at 6 h after 5 °C treatment. Of them, expression levels of five *CsTCPs* (*CsTCP1*, − *3*, − *4*, − *7*, − *12*) were significantly fluctuated along with the extension of 5 °C treatment; the expression level of one *CsTCP* (*CsTCP9*) was just significantly increased at 6 h after 5 °C treatment. However, transcript levels of eight *CsTCPs* (*CsTCP5*, − *8*, − *11*, − *13*, − *15*, − *16*, − *17*, − *20*) presented contrary expression trends, namely, were increased at 2 h after 5 °C treatment and then were decreased at 6 h after 5 °C treatment. Of them, expression levels of six *CsTCPs* (*CsTCP5*, − *8*, − *13*, − *15*, − *17*, − *20*) were significantly fluctuated along with the extension of 5 °C treatment; the expression level of one *CsTCP* (*CsTCP16*) was just significantly decreased at 6 h after 5 °C treatment. On the other hand, transcript levels of six *CsTCPs* (*CsTCP2*, − *6*, − *10*, − *14*, − *18*, − *19*) were up-regulated after 5 °C treatments. Of them, expression levels of three *CsTCPs* (*CsTCP2*, − *6*, − *14*) were significantly increased after 5 °C treatments; the expression level of one *CsTCP* (*CsTCP10*) was significantly increased at 2 h after 5 °C treatment and then was almost kept stable at 6 h after 5 °C treatment; the expression level of one *CsTCP* (*CsTCP18*) was just significantly increased at 6 h after 5 °C treatment.

In addition, the effect of drought on expression patterns of twenty *CsTCPs* were also investigated (Fig. 5C). After drought treatment, the soil water content was significantly decreased and the proline content in mature leaf was significantly increased (Fig. S2B, C); transcript levels of seven *CsTCPs* were also significantly influenced. Of them, expression levels of two *CsTCPs* (*CsTCP11*, − *12*) exhibited significant increase and were about 1.4- and 7.3-fold higher than those in the control, respectively; by contrast, expression levels of five *CsTCPs* (*CsTCP14*, − *16*, − *18*, − *19*, − *20*) exhibited significant decrease and were about 0.6, 0.2, 0.4, 0.4, and 0.2 times of those in the control, respectively. Moreover, expression levels of two *CsTCPs* (*CsTCP15*, − *17*) were reduced to less than half of those in the control (Fig. 5C).

## Discussion

Plant-specific TCP family, widely distributed in plants but with different numbers [3, 4, 11, 32], is well known as a group of transcription factors to regulate plant growth and development; its members contain a highly conserved TCP domain with about 59 amino acids at the N-terminus [2]. Moreover, TCP members are generally divided into three groups, including PCF, CIN, and CYC/TB1 subclades, based on the homology and variation of TCP domain [5, 6]. In this study, a total of twenty putative TCP members with the TCP domain were explored from *Citrus sinensis* genome by systematical alignment and screening (Table 1, Fig. 1B); they were divided into three subclades, of which eleven, five, and four members belonged to subclades PCF, CIN, and CYC/TB1, respectively (Fig. 1A, B). These results suggested that there are at least twenty *TCP* genes in *Citrus sinensis* genome. In general, duplicated genes are considered to be paralogs forming a gene family, and they are thought to provide raw materials for the generation of new genes, which can facilitate the generation of new functions in turn [33]. As found in twenty *CsTCPs*, six pairs of paralogous genes, including *CsTCP3* and *CsTCP11, CsTCP5* and *CsTCP10, CsTCP6* and *CsTCP18, CsTCP7* and *CsTCP9, CsTCP8* and *CsTCP12,* as well as *CsTCP12* and *CsTCP14* (Fig. 3A), suggested that the segmental duplication may contribute to the amplification of *TCP* gene family in *Citrus sinensis* genome. In addition, the *K*a/*K*s ratio of each pair was far lower than 1 (Table S1). Based on the viewpoint of the previous study [34], the purifying selection may play a major role in the evolution of *CsTCPs*.

To date, *TCP* genes have been reported to function largely in many aspects of plant growth and development [2]. Gene expression is a biological process by which the genetic information in DNA is converted to mRNA and then translated to protein, namely, gene function is eventually performed in form of protein [35]. Herein, physical and chemical properties of proteins, gene structure, and motif composition of *Citrus sinensis* TCP members were different even in the same subclade (Table 1, Fig. S1), suggesting that twenty *CsTCPs* may have different functions. The functions of some *CsTCPs* were discussed in the following.

*CsTCP3*, a *PCF*-type gene, was relead to *AtTCP20* in phylogenetic relationships and was the ortholog of *AtTCP20* (Fig. 1A, Fig. 3B). Previous study indicated that *AtTCP20* can regulate leaf development via the jasmonate signalling pathway, especially during early leaf developmental stages in *Arabidopsis thaliana* [13]. Given the viewpoint that colinear genes in relative species contain a lot of homologous functions [36], *CsTCP3* might have functions similar to *AtTCP20*. Indeed, the highest transcript level of *CsTCP3* was observed in leaf and two MeJA-responsive elements were found in its promoter region (Fig. 2A, Fig. 4B). These results suggested that *CsTCP3* may also participate in leaf development. Moreover, *CsTCP15* was another PCF subclade member and was orthologous to *AtTCP11* (Fig. 1A, Fig. 3B). Previous study demonstrated that *AtTCP11* can influence the development of leaf, stem, petiole, and pollen in *Arabidopsis thaliana* [20]. The present study indicated that transcript levels of *CsTCP15* in stem, mature leaf, and old thorn were higher than those in other tissues or organs (Fig. 4B), and *cis*-acting elements, such as gibberellin (GA)- and ABA-responsive elements, were found in its promoter region (Fig. 2A), further suggesting that *CsTCP15* possibly functions in the development of stem, leaf, or thorn (Fig. 4B).

*CsTCP9*, belonging to CIN subclade, was closely related to *AtTCP13* in phylogenetic relationships (Fig. 1A). *AtTCP13* is a regulator mediating leaf development in *Arabidopsis thaliana* [14]. Herein, *CsTCP9* was the ortholog of *AtTCP13* and was highly expressed in leaf (Fig. 3B, Fig. 4B), and it contained some phytohormone-reponsive elements (Fig. 2A), suggesting that *CsTCP9* may be involved in regulating leaf development. On the other hand, another *CIN*-type gene, *CsTCP13* was closely related to *AtTCP4* in phylogenetic relationships and was orthologous to *AtTCP4* (Fig. 1A, Fig. 3B). *AtTCP4* was confirmed to modulate cell proliferation at margins of the developing leaf in *Arabidopsis thaliana* by antagonizing *miR319* [27, 28], and its ortholog *LsTCP4* can participate in affecting the leaf shape phenotype of *Lactuca sativa* [37]. Notably, *CsTCP13* also contained the *miR319*-binding site (Fig. 1D). The highest transcript level of *CsTCP13* was observed in leaf and some *cis*-acting elements related to phytohormones were found in its promoter region (Fig. 2A, Fig. 4B). Hence, these results suggested that *CsTCP13* possibly takes part in leaf development by antagonizing *miR319*. In addtion, homodimers and heterodimers can be formed among TCP proteins, and these oligomerization combinations possess different affinity to bind various DNA components to regulate plant growth and development [5]. Herein, the protein-protein interaction network showed that CsTCP13 interacted with many other TCP members (Fig. 2B), suggesting that CsTCP13 probably plays an important role in leaf development presumably by forming protein complexes.

Shoot branching determines plant architecture, which is essential to maintain yeild in many crops [38]. *AtTCP18* (*BRC1*) and *AtTCP12* (*BRC2*), belonging to CYC/TB1 subclade, were confirmed to control axillary bud outgrowth in *Arabidopsis thaliana* [11]; especially, *BRC1* and its orthologs in many species are generally regarded as an integrator of branching signals regulating bud outgrowth [39]. In this study, two *CYC/TB1*-type genes, *CsTCP14* and *CsTCP12* were closely related to *BRC1* and *BRC2* in phylogenetic relationships, respectively (Fig. 1A), and collinearity analysis indicated that they were orthologous to *BRC1* and *BRC2*, respectively (Fig. 3B). Moreover, *CsTCP14* and *CsTCP12* were highly expressed in bud, and they both contained some phytohormone-reponsive elements (Fig. 2A, Fig. 4B). These results suggested that *CsTCP14* and *CsTCP12* may function in shoot branching, similar to *BRC1* and *BRC2*, respectively. On the other hand, *TI1* (the ortholog of *BRC1* in citrange) is required in thorn conversion, and *PcBRC2* (the ortholog of *BRC2* in poplar) was confirmed to play a key role in leaf development [12, 40]. The present study found that the highest transcript levels of *CsTCP14* and *CsTCP12* were observed in thorn and leaf, respectively (Fig. 4B), and they were fluctuated with the development of thorn and leaf, respectively (Fig. 4B), suggesting that *CsTCP14* and *CsTCP12* may participate in regulating the development of thorn and leaf, respectively.

Low intensity of light, abnormal temperature, and drought are three abiotic stresses that plants endure frequently in the process of growth and development [41]. Previous reports indicated that *TCP* genes can be involved in response to abiotic stresses. For example, *BRC1* was confirmed to promote axillary bud dormancy responding to shading in *Arabidopsis thaliana* [42]; *OsPCF6* and *OsTCP21* were found to influence the sensitivity to low temperature in *Oryza sativa* [43]; moreover, *ZmTCP32* and *ZmTCP42* were confirmed to associate with drought tolerance, and *ZmTCP42* acted as a positive regulator responding to drought in *Zea mays* [44]. In this study, expression levels of *CsTCP3* and *CsTCP15* were significantly fluctuated in mature leaf under low temperature (Fig. 5B), and low temperature-reponsive elements were found in their promoter regions (Fig. 2A), suggesting that *CsTCP3* and *CsTCP15* may take part in the response of leaf to low temperature. On the other hand, *CsTCP9* and *CsTCP13* both contained at least five light-responsive elements (Fig. 2A), and their expression levels were significantly reduced in mature leaf by shading (Fig. 5A), suggesting that *CsTCP9* and *CsTCP13* are possibly involved in response to shading besides the regulation of leaf development. In addition, *CsTCP12* contained ABA- and drought-responsive elements, and its expression level was significantly increased in mature leaf by drought; *CsTCP14* contained seven ABA-responsive elements, but its expression level in mature leaf was significantly decreased by drought (Fig. 2A, Fig. 5C); moreover, the opposite trend in the change of their expression levels was also observed in mature leaf by shading and low temperature (Fig. 5A, B). These results suggested that these two genes may function differently in the response of leaf to shading, low temperature, and drought, and are worthy of further study in the future.

## Conclusions

In this study, twenty putative *CsTCPs* with the TCP domain were explored from *Citrus sinensis* genome, of which eleven, five, and four *CsTCPs* were clustered into subclades PCF, CIN, and CYC/TB1, respectively. The segmental duplication may promote the amplification of *TCP* gene family in *Citrus sinensis* genome, and the purifying selection majorly contributes to the evolution of *CsTCPs*. The twenty *CsTCPs* may have their own functions due to their different protein properties, gene structure, motif composition, and their varied expression profiles in tissues or organs, as well as in response to abiotic stresses. *CsTCP3*, *CsTCP9*, and *CsTCP13* are probably involved in the regulation of leaf development; specially, *CsTCP13* may perform its function by antagonizing *miR319* or by forming protein complexes. *CsTCP12* and *CsTCP14* possibly function in shoot branching; specially, *CsTCP12* may also act as a regulator in leaf development, and *CsTCP14* may also play an important role in thorn development. *CsTCP15* may take part in the development of stem, leaf, or thorn. *CsTCP3* and *CsTCP15*, *CsTCP9* and *CsTCP13*, as well as *CsTCP12* and *CsTCP14* are probably involved in the response of leaf to low temperature, shading, and drought, respectively. Altogether, the present results suggested possible roles of some *TCP* genes, and their specific roles and potential mechanisms during citrus plant growth and development as well as in response to abiotic stresses are required to be further studied in the future.

## Methods

### Plant materials

Roots, stems, leaves, thorns, and buds were collected from two-year-old seedlings of *Poncirus trifoliata*. In detail, roots included primary roots and lateral roots; stems included tip stems (non-lignified), middle stems (semi-lignified), and base stems (lignified); leaves included young leaves (3 weeks old), mature leaves (3 months old), and old leaves (6 months old) of autumn shoots; thorns or buds included young thorns or young buds (from non-lignified shoots) and old thorns or old buds (from lignified shoots). In this study, fifteen healthy seedlings were randomly selected and divided into three groups as three biological replicates for sample collection. Moreover, flowers were harvested at full flowering stage, as well as fruits and seeds were harvested at 190 days after flowering (DAF) from adult trees of *Poncirus trifoliata*. In addition, *Citrus reticulata* cv. Kinokuni adult trees grafted on *Poncirus trifoliata* were used to investigate the expression patterns of *TCP* genes responding to shade, low temperature, and drought treatments.

All the plant materials were located in citrus germplasm orchard of Huazhong Agricultural University (Wuhan, Hubei Province, China). Harvested samples were rapidly frozen by liquid nitrogen and immediately stored at − 80 °C.

### Abiotic stress treatments

Shade and drought treatments were applied to adult trees of *Citrus reticulata* cv. Kinokuni at 135 DAF. For shade treatment, three healthy adult trees were randomly selected as three biological replicates. On each tree, two robust branches at the top of the same crown were regarded as one comparison. Of them, one branch was covered with a black shading net which transmits about 10% of incident light [45]; the other branch was not covered as the control. The light intensity of crown was measured by digital illuminance meter (GM1010; BENETECH, Shenzhen, China) at 14:00 and 18:00 on a sunny day. One week later, healthy mature leaves from the third to the sixth node of spring shoots were collected.

For drought treatment, six healthy adult trees were randomly selected and the soil was covered by black plastic films. Of them, three trees as three biological replicates were irrigated once per week (20 L of water per tree) as the control [46]; the three other trees were not irrigated. After 2 weeks, soil water content at 30 cm below the surface was detected by the oven-drying method [47], and healthy mature leaves from the third to the sixth node of spring shoots were collected. The proline content of mature leaves was determined by Proline Assay Kit (vis-spectrophotometry; Solarbio, Beijing, China). On the other hand, healthy mature leaves without shade and drought treatments were stored together with the shoots at 5 °C for zero hour (Control), 2 h (T1), and 6 h (T2), respectively. Each treatment contained at least 30 leaves. Then, all the collected samples were rapidly frozen by liquid nitrogen and immediately stored at − 80 °C.

### Identification of *TCP* genes from *Citrus sinensis* genome

The complete genome sequence data of *Citrus sinensis* v1.0 were downloaded from Citrus Pan-genome to Breeding Database (http://citrus.hzau.edu.cn/). The protein sequences of AtTCP transcription factors were retrieved from The Arabidopsis Information Resource (https://www.arabidopsis.org/). Based on such two files, the two-step BLAST method was used to explore *TCP* genes from *Citrus sinensis* genome with the Blast Compare Two Seqs program of TBtools [48]. In detail, AtTCPs were used as query sequences to search all possible CsTCPs (e-value, 1e-10) from subject sequences, which were translated by representative mRNA sequences from *Citrus sinensis* genome. Moreover, the Hidden Markov Model profile of TCP domain (PF03634) retrieved from Pfam database (http://pfam.xfam.org/) was used as the standard; all candidate CsTCPs were further screened out according to this standard by applying the phmmer program (https://www.ebi.ac.uk/Tools/hmmer/search/phmmer), the hmmscan program (https://www.ebi.ac.uk/Tools/hmmer/search/hmmscan), and Batch Web CD-Search Tool (https://www.ncbi.nlm.nih.gov/Structure/bwrpsb/bwrpsb.cgi).

### Gene location, duplication, structure, and characterization

The genomic distribution of putative *CsTCPs* on chromosomes, the chromosomal repeat fragment information, and gene structure were visualized by the Advanced Circos program and the Gene Structure View (Advanced) program of TBtools, respectively. The length of coding sequences (CDS), the size of proteins, and the *K*a/*K*s ratio were calculated by the Fasta Stats program and the Simple Ka/Ks Calculator (NG) program of TBtools, respectively; the *K*a/*K*s ratio was used to analyze the trend of gene divergence after duplication events with the criteria that *K*a/*K*s < 1 means the purifying selection, *K*a/*K*s = 1 means the neutral selection, and *K*a/*K*s > 1 means the positive selection leading to the accelerated evolution [34].

In addition, Mw and theoretical pI of CsTCPs were computed by the Compute pI/Mw tool (https://web.expasy.org/compute_pi/); the length of signal peptide was calculated with the SignalP-5.0 server (http://www.cbs.dtu.dk/services/SignalP/); the predicted subcellular location information was retrieved with CELLO v.2.5 (http://cello.life.nctu.edu.tw/).

### Phylogenetic analysis, conserved domain identification, and *miR319*-binding site recognition

The protein sequences of CsTCPs, AtTCPs, and SlTCPs [32] were used to construct phylogenetic tree by the neighbor-joining method with MEGA X (https://www.megasoftware.net/) and iTOL v.6 (https://itol.embl.de/); the bootstrap test was implemented with 1000 iterations.

On the other hand, the protein sequences of CsTCPs and AtTCPs were aligned by the Muscle method with MEGA X, and the overall conserved amino acids were visualized with Jalview v.2.11.1.4 (http://www.jalview.org/). Moreover, *miR319*-binding sites of *CsTCPs* were predicted by psRNATarget (https://www.zhaolab.org/psRNATarget/).

### Analysis of conserved motif, *cis*-acting element, collinearity relationship, GO annotation, and protein-protein interaction

The conserved motif composition of CsTCP protein sequences was analyzed by online program MEME v.5.4.1 (https://meme-suite.org/meme/tools/meme) and visualized by the Gene Structure View (Advanced) program of TBtools. *cis*-acting elements in promoter regions of 2 kb upstream of translation initiation sites of *CsTCPs* were screened out in PlantCARE (http://bioinformatics.psb.ugent.be/webtools/plantcare/html/).

Moreover, the collinearity relationships between *CsTCPs* and *AtTCPs* were analyzed by the One Step MCScanX-Super Fast program of TBtools. The GO annotations of *CsTCPs* were performed with EGGNOG-Mapper (http://eggnog-mapper.embl.de/) and visualized by WEGO v.2.0 (https://wego.genomics.cn/). The protein-protein interaction network of CsTCPs was constructed by STRING v.11.5 (https://string-db.org/) and Cytoscape v.3.6.1 (https://cytoscape.org/).

### RNA extraction and quantitative real-time PCR (qRT-PCR)

Total RNA of each sample was extracted by OminiPlant RNA Kit (CWBIO, Beijing, China). One microgram (μg) of high-quality total RNA was used for the first-strand cDNA synthesis by *TransScript* One-step gDNA Removal and cDNA Synthesis SuperMix (TransGen Biotech, Beijing, China). The qRT-PCR was conducted with three biological replicates, and each biological replicate was technically performed for three times in a 10 μL reaction volume using Hieff qPCR SYBR Green Master Mix (YEASEN, Shanghai, China) on the QuantStudio™ 6 Flex Real-Time PCR System (Thermo Fisher Scientific, USA). The reaction started with 95 °C for 5 min, then followed by 40 cycles of 95 °C for 10 s, 60 °C for 20 s and 72 °C for 20 s. In this study, *CsActin* (Gene ID: Cs1g05000.1) was used as the internal control, and specific primers of target genes for qRT-PCR were designed by Primer Premier 5 (http://www.premierbiosoft.com/primerdesign/) and listed in Table S3. The relative mRNA expression values were calculated with the Livak method [49].

### Statistical analysis

The data were analyzed by *t*-test or by Tukey test in ANOVA program of IBM SPSS Statistics v.26 (https://www.ibm.com/cn-zh/analytics/spss-statistics-software); the level of significance was set at *P* < 0.05. The graphs were created by SigmaPlot v.12.5 (https://systatsoftware.com/products/sigmaplot/), RStudio (https://rstudio.com/), or TBtools.

## Supplementary Information

**Additional file 1: Fig. S1.** Motif composition and gene structure of *Citrus sinensis* TCP members. A total of twenty motifs in *Citrus sinensis* TCP protein sequences were analyzed by MEME algorithm v.5.4.1. The structure of twenty TCP genes was visualized by the Gene Structure View (Advanced) program of TBtools. Different motifs, coding sequences (CDS), and untranslated regions (UTR) are represented by colored boxes. Introns are represented by gray lines. Tick labels represent protein length (aa) and gene length (bp).

**Additional file 2: Table S1.** Ka/Ks of TCP gene pairs in *Citrus sinensis* genome.

**Additional file 3: Table S2.** Gene ontology (GO) of *Citrus sinensis* TCP genes.

**Additional file 4: Fig. S2.** Illustration of differential conditions under shade and drought treatments. **A.** Light intensity in the canopy under shade treatment. The data were collected by digital illuminance meter at 14:00 and 18:00 on a sunny day. **B.** Absolute rate of water to soil under drought treatment. **C.** Proline content in mature leaf of *Citrus reticulata* cv. Kinokuni under drought treatment. Results are the mean of three independent biological replicates. Error bars represent the standard deviation of replicates. The asterisk indicates statistically significant difference between groups at *P* < 0.05 by t-test.

**Additional file 5: Table S3.** Primers for quantitative real-time PCR (qRT-PCR).

**Additional file 6: Table S4.** List of accession IDs or numbers of all sequences used in this study.

## Data Availability

The whole genome data of *Citrus sinensis* v1.0 were downloaded from Citrus Pan-genome to Breeding Database (http://citrus.hzau.edu.cn/), and the published TCP sequences of *Arabidopsis thaliana* and *Solanum lycopersicum* were acquired from The Arabidopsis Information Resource database (https://www.arabidopsis.org/) and *Solanum lycopersicum* ITAG2.4 of Phytozome genome database (https://phytozome-next.jgi.doe.gov/), respectively. The accession IDs or numbers of all sequences used in the present study are listed in Table S4, and all databases used in this study are available to the public. All other data are contained within the article or its supplementary information, and they are available upon reasonable request.

## Abbreviations

aa: Amino acid(s)
ABA: Abscisic acid
CDS: Coding sequence(s)
CIN: CINCINNATA
CYC/TB1: CYCOLOIDEA and TEOSINTE BRANCHED1
Da: Dalton(s)
DAF: Days after flowering
GA: Gibberellin
GO: Gene ontology
*K*a: Nonsynonymous substitution rate
*K*s: Synonymous substitution rate
MeJA: Methyl jasmonate
Mw: Molecular weight
PCF: PROLIFERATING CELL FACTORS
pI: Isoelectric point
qRT-PCR: Quantitative real-time PCR
SA: Salicylic acid
TCP: TEOSINTE BRANCHED1, CYCOLOIDEA, and PROLIFERATING CELL FACTORS
*UTR*: Untranslated region(s)
μg: Microgram(s)

## References

[1] Katagiri F, Chua N-H (1992). Plant transcription factors: present knowledge and future challenges. Trends Genet.

[2] Martín-Trillo M, Cubas P (2010). TCP genes: a family snapshot ten years later. Trends Plant Sci.

[3] Floyd Sandra K, Bowman JL (2007). The ancestral developmental tool kit of land plants. Int J Plant Sci.

[4] Navaud O, Dabos P, Carnus E, Tremousaygue D, Hervé C (2007). TCP transcription factors predate the emergence of land plants. J Mol Evol.

[5] Kosugi S, Ohashi Y (2002). DNA binding and dimerization specificity and potential targets for the TCP protein family. Plant J.

[6] Manassero NGU, Viola IL, Welchen E, Gonzalez DH (2013). TCP transcription factors: architectures of plant form. BioMolecular Concepts.

[7] Cubas P, Lauter N, Doebley J, Coen E (1999). The TCP domain: a motif found in proteins regulating plant growth and development. Plant J.

[8] Davière J-M, Wild M, Regnault T, Baumberger N, Eisler H, Genschik P, Achard P (2014). Class I TCP-DELLA interactions in Inflorescence shoot apex determine plant height. Curr Biol.

[9] Shi P, Guy KM, Wu W, Fang B, Yang J, Zhang M, Hu Z (2016). Genome-wide identification and expression analysis of the ClTCP transcription factors in Citrullus lanatus. BMC Plant Biol.

[10] Takeda T, Suwa Y, Suzuki M, Kitano H, Ueguchi-Tanaka M, Ashikari M, Matsuoka M, Ueguchi C (2003). The OsTB1 gene negatively regulates lateral branching in rice. Plant J.

[11] Aguilar-Martínez JA, Cs P-C, Cubas P (2007). Arabidopsis BRANCHED1 acts as an integrator of branching signals within axillary buds. Plant Cell.

[12] Zhang F, Rossignol P, Huang T, Wang Y, May A, Dupont C, Orbovic V, Irish VF (2020). Reprogramming of stem cell activity to convert thorns into branches. Curr Biol.

[13] Danisman S, van der Wal F, Dhondt S, Waites R, de Folter S, Bimbo A, van Dijk AD, Muino JM, Cutri L, Dornelas MC (2012). Arabidopsis class I and class II TCP transcription factors regulate Jasmonic acid metabolism and leaf development antagonistically. Plant Physiol.

[14] Hur Y-S, Kim J, Kim S, Son O, Kim W-Y, Kim G-T, Ohme-Takagi M, Cheon C-I (2019). Identification of TCP13 as an upstream regulator of ATHB12 during leaf development. Genes..

[15] Vadde BVL, Challa KR, Nath U (2018). The TCP4 transcription factor regulates trichome cell differentiation by directly activating GLABROUS INFLORESCENCE STEMS in Arabidopsis thaliana. Plant J.

[16] Lan J, Zhang J, Yuan R, Yu H, An F, Sun L, Chen H, Zhou Y, Qian W, He H (2021). TCP transcription factors suppress cotyledon trichomes by impeding a cell differentiation-regulating complex. Plant Physiol.

[17] Luo D, Carpenter R, Vincent C, Copsey L, Coen E (1996). Origin of floral asymmetry in Antirrhinum. Nature..

[18] Wang J, Wang Y, Luo D (2010). LjCYC genes constitute floral Dorsoventral asymmetry in Lotus japonicus. J Integr Plant Biol.

[19] Takeda T, Amano K, Ohto M-a, Nakamura K, Sato S, Kato T, Tabata S, Ueguchi C (2006). RNA interference of the Arabidopsis putative transcription factor TCP16 gene results in abortion of early pollen development. Plant Mol Biol.

[20] Viola IL, Uberti Manassero NG, Ripoll R, Gonzalez DH (2011). The Arabidopsis class I TCP transcription factor AtTCP11 is a developmental regulator with distinct DNA-binding properties due to the presence of a threonine residue at position 15 of the TCP domain. Biochem J.

[21] Tatematsu K, Nakabayashi K, Kamiya Y, Nambara E (2008). Transcription factor AtTCP14 regulates embryonic growth potential during seed germination in Arabidopsis thaliana. Plant J.

[22] Resentini F, Felipo-Benavent A, Colombo L, Blázquez MA, Alabadí D, Masiero S (2015). TCP14 and TCP15 mediate the promotion of seed germination by gibberellins in Arabidopsis thaliana. Mol Plant.

[23] Zhang W, Cochet F, Ponnaiah M, Lebreton S, Matheron L, Pionneau C, Boudsocq M, Resentini F, Huguet S, Blázquez MÁ (2019). The MPK8-TCP14 pathway promotes seed germination in Arabidopsis. Plant J.

[24] Giraud E, Ng S, Carrie C, Duncan O, Low J, Lee CP, Van Aken O, Millar AH, Murcha M, Whelan J (2010). TCP transcription factors link the regulation of genes encoding mitochondrial proteins with the circadian clock in Arabidopsis thaliana. Plant Cell.

[25] Braun N, de Saint GA, Pillot J-P, Boutet-Mercey S, Dalmais M, Antoniadi I, Li X, Maia-Grondard A, Le Signor C, Bouteiller N (2011). The pea TCP transcription factor PsBRC1 acts downstream of Strigolactones to control shoot branching. Plant Physiol.

[26] Fang Y, Zheng Y, Lu W, Li J, Duan Y, Zhang S, Wang Y (2021). Roles of miR319-regulated TCPs in plant development and response to abiotic stress. Crop J.

[27] Bresso EG, Chorostecki U, Rodriguez RE, Palatnik JF, Schommer C (2017). Spatial control of gene expression by miR319-regulated TCP transcription factors in leaf development. Plant Physiol.

[28] Schommer C, Debernardi JM, Bresso EG, Rodriguez RE, Palatnik JF (2014). Repression of cell proliferation by miR319-regulated TCP4. Mol Plant.

[29] Liu Y, Heying E, Tanumihardjo SA (2012). History, global distribution, and nutritional importance of Citrus fruits. Compr Rev Food Sci Food Saf.

[30] Goodstein DM, Shu S, Howson R, Neupane R, Hayes RD, Fazo J, Mitros T, Dirks W, Hellsten U, Putnam N (2011). Phytozome: a comparative platform for green plant genomics. Nucleic Acids Res.

[31] Xu Q, Chen L-L, Ruan X, Chen D, Zhu A, Chen C, Bertrand D, Jiao W-B, Hao B-H, Lyon MP (2013). The draft genome of sweet orange (Citrus sinensis). Nat Genet.

[32] Parapunova V, Busscher M, Busscher-Lange J, Lammers M, Karlova R, Bovy AG, Angenent GC, de Maagd RA (2014). Identification, cloning and characterization of the tomato TCP transcription factor family. BMC Plant Biol.

[33] Zhang J (2003). Evolution by gene duplication: an update. Trends Ecol Evol.

[34] Hurst LD (2002). The Ka/Ks ratio: diagnosing the form of sequence evolution. Trends Genet.

[35] Uzman A (2007). Fundamental molecular biology. Biochem Mol Biol Educ.

[36] Angiuoli SV, Salzberg SL (2010). Mugsy: fast multiple alignment of closely related whole genomes. Bioinformatics..

[37] Seki K, Komatsu K, Tanaka K, Hiraga M, Kajiya-Kanegae H, Matsumura H, Uno Y (2020). A CIN-like TCP transcription factor (LsTCP4) having retrotransposon insertion associates with a shift from Salinas type to empire type in crisphead lettuce (Lactuca sativa L.). horticulture. Research..

[38] Teichmann T, Muhr M (2015). Shaping plant architecture. Front Plant Sci.

[39] Barbier FF, Dun EA, Kerr SC, Chabikwa TG, Beveridge CA (2019). An update on the signals controlling shoot branching. Trends Plant Sci.

[40] Muhr M, Paulat M, Awwanah M, Brinkkötter M, Teichmann T (2018). CRISPR/Cas9-mediated knockout of Populus BRANCHED1 and BRANCHED2 orthologs reveals a major function in bud outgrowth control. Tree Physiol.

[41] Gong Z, Xiong L, Shi H, Yang S, Herrera-Estrella LR, Xu G, Chao D-Y, Li J, Wang P-Y, Qin F (2020). Plant abiotic stress response and nutrient use efficiency. Sci China Life Sci.

[42] González-Grandío E, Poza-Carrión C, Sorzano COS, Cubas P (2013). BRANCHED1 promotes axillary bud dormancy in response to shade in Arabidopsis. Plant Cell.

[43] Wang S-t, Sun X-l, Hoshino Y, Yu Y, Jia B, Sun Z-w, Sun M-z, Duan X-b, Zhu Y-m (2014). MicroRNA319 positively regulates cold tolerance by targeting OsPCF6 and OsTCP21 in rice (Oryza sativa L.). PLoS One.

[44] Ding S, Cai Z, Du H, Wang H (2019). Genome-wide analysis of TCP family genes in Zea mays L. identified a role for ZmTCP42 in drought tolerance. Int J Mol Sci.

[45] Yang X-Y, Xie J-X, Lu X-P, Liu Y-Z, Peng S-A (2011). Isolation of a citrus ethylene-responsive element binding factor gene and its expression in response to abiotic stress, girdling and shading. Sci Hortic.

[46] Jiang N, Jin L-F, Teixeira da Silva JA, Islam MDZ, Gao H-W, Liu Y-Z, Peng S-A (2014). Activities of enzymes directly related with sucrose and citric acid metabolism in citrus fruit in response to soil plastic film mulch. Sci Hortic.

[47] Jin L-F, Guo D-Y, Ning D-y, Hussain SB, Liu Y-Z (2018). Covering the trees of Kinokuni tangerine with plastic film during fruit ripening improves sweetness and alters the metabolism of cell wall components. Acta Physiol Plant.

[48] Chen C, Chen H, Zhang Y, Thomas HR, Frank MH, He Y, Xia R (2020). TBtools: An integrative toolkit developed for interactive analyses of big biological data. Mol Plant.

[49] Livak KJ, Schmittgen TD (2001). Analysis of relative gene expression data using real-time quantitative PCR and the 2−ΔΔCT method. Methods..

