# Supplementary material for: Genome-wide analysis of citrus TCP transcription factors and their responses to abiotic stresses
Source: BMC Plant Biol. 2022 Jul 6;22:325. doi: 10.1186/s12870-022-03709-3 (PMC9258177; doi:10.1186/s12870-022-03709-3)
Supplement: Supplementary file 1 — Additional file 1: Fig. S1. Motif composition and gene structure of Citrus sinensis TCP members. A total of twenty motifs in Citrus sinensis TCP protein sequences were analyzed by MEME algorithm v.5.4.1. The structure of twenty TCP genes was visualized by the Gene Structure View (Advanced) program of TBtools. Different motifs, coding sequences (CDS), and untranslated regions (UTR) are represented by colored boxes. Introns are represented by gray lines. Tick labels represent protein length (aa) and gene length (bp). [file 12870_2022_3709_MOESM1_ESM.docx]

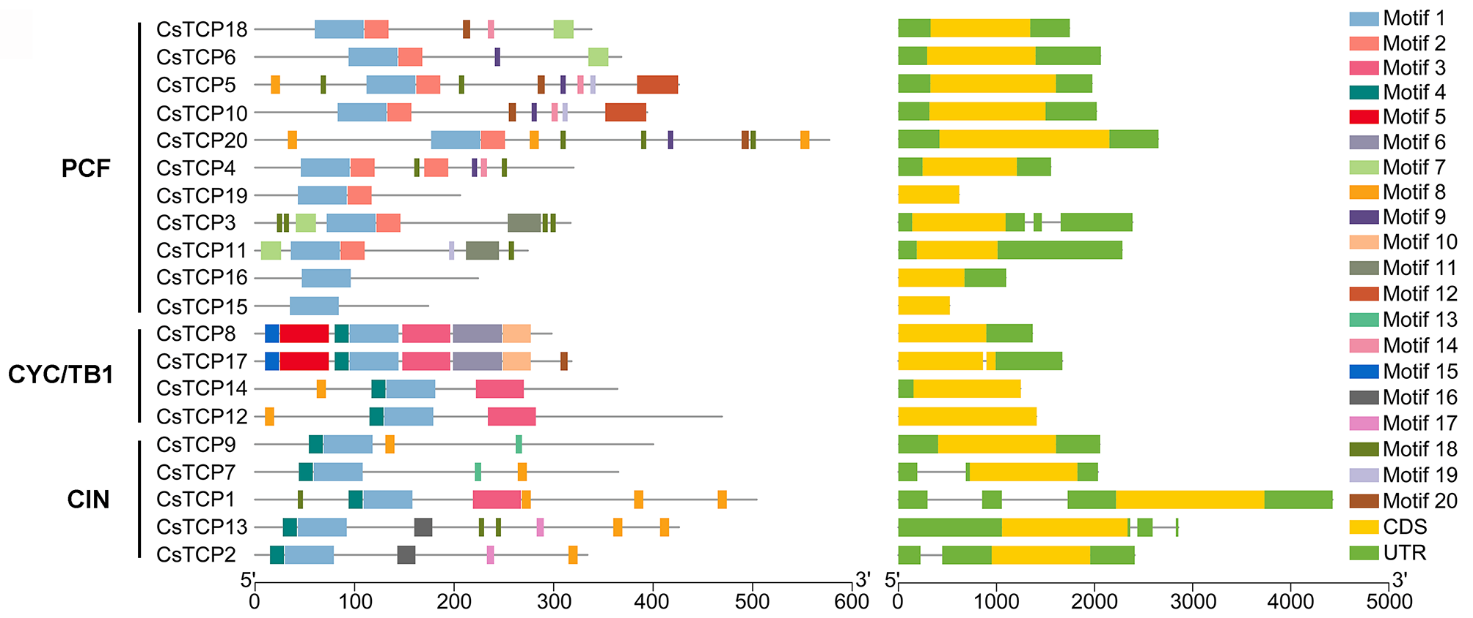


**Fig. S1** Motif composition and gene structure of *Citrus sinensis* TCP members. A total of twenty motifs in *Citrus sinensis* TCP protein sequences were analyzed by MEME algorithm v.5.4.1. The structure of twenty *TCP* genes was visualized by the Gene Structure View (Advanced) program of TBtools. Different motifs, coding sequences (CDS), and untranslated regions (*UTR*) are represented by colored boxes. Introns are represented by gray lines. Tick labels represent protein length (aa) and gene length (bp)
