# Supplementary material for: Genome-wide analysis of citrus TCP transcription factors and their responses to abiotic stresses
Source: BMC Plant Biol. 2022 Jul 6;22:325. doi: 10.1186/s12870-022-03709-3 (PMC9258177; doi:10.1186/s12870-022-03709-3)
Supplement: Supplementary file 2 — Additional file 2: Table S1. Ka/Ks of TCP gene pairs in Citrus sinensis genome. [file 12870_2022_3709_MOESM2_ESM.docx]

**Table S1** *K*a/*K*s of *TCP* gene pairs in *Citrus sinensis* genome

| Gene pairs | *K*a | *K*s | *K*a/*K*s |
| --- | --- | --- | --- |
| *CsTCP3-CsTCP11* | 0.234563629 | 1.902302382 | 0.123305123 |
| *CsTCP5-CsTCP10* | 0.340974487 | 1.811327774 | 0.188245602 |
| *CsTCP6-CsTCP18* | 0.434025267 | 3.249103174 | 0.133583098 |
| *CsTCP7-CsTCP9* | 0.530728023 | 1.878760006 | 0.282488461 |
| *CsTCP8-CsTCP12* | 0.606008732 | 2.217152866 | 0.273327447 |
| *CsTCP12-CsTCP14* | 0.596317654 | 2.405045139 | 0.247944475 |

*K*a, nonsynonymous substitution rate; *K*s, synonymous substitution rate
