# Supplementary material for: Genome-wide analysis of citrus TCP transcription factors and their responses to abiotic stresses
Source: BMC Plant Biol. 2022 Jul 6;22:325. doi: 10.1186/s12870-022-03709-3 (PMC9258177; doi:10.1186/s12870-022-03709-3)
Supplement: Supplementary file 4 — Additional file 4: Fig. S2. Illustration of differential conditions under shade and drought treatments. A. Light intensity in the canopy under shade treatment. The data were collected by digital illuminance meter at 14:00 and 18:00 on a sunny day. B. Absolute rate of water to soil under drought treatment. C. Proline content in mature leaf of Citrus reticulata cv. Kinokuni under drought treatment. Results are the mean of three independent biological replicates. Error bars represent the standard deviation of replicates. The asterisk indicates statistically significant difference between groups at P < 0.05 by t-test. [file 12870_2022_3709_MOESM4_ESM.docx]

­
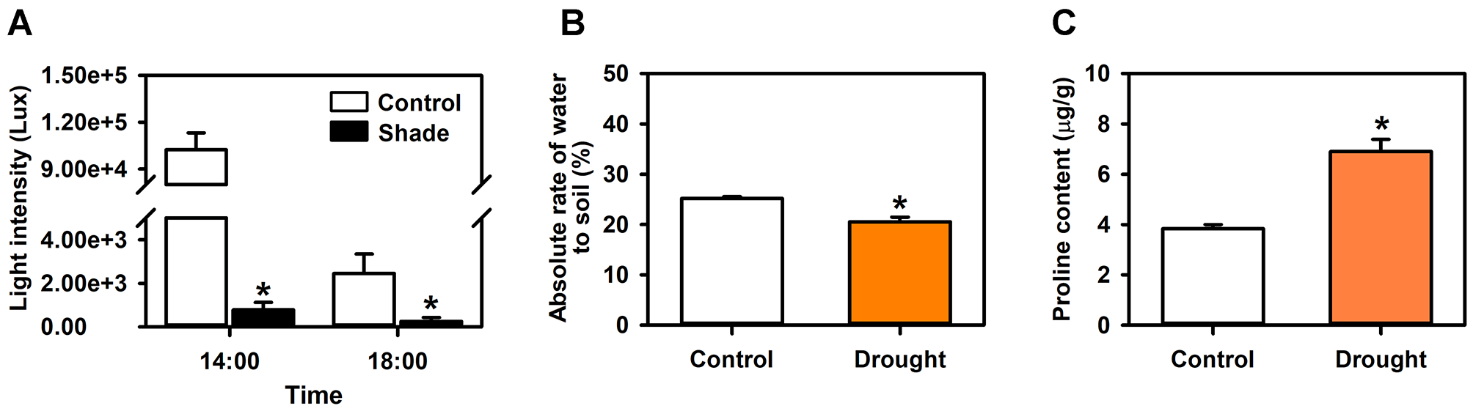


**Fig. S2** Illustration of differential conditions under shade and drought treatments. **A.** Light intensity in the canopy under shade treatment. The data were collected by digital illuminance meter at 14:00 and 18:00 on a sunny day. **B.** Absolute rate of water to soil under drought treatment. **C.** Proline content in mature leaf of *Citrus reticulata* cv. Kinokuni under drought treatment. Results are the mean of three independent biological replicates. Error bars represent the standard deviation of replicates. The asterisk indicates statistically significant difference between groups at *P* < 0.05 by *t*-test
