# Supplementary material for: Genome-wide analysis of citrus TCP transcription factors and their responses to abiotic stresses
Source: BMC Plant Biol. 2022 Jul 6;22:325. doi: 10.1186/s12870-022-03709-3 (PMC9258177; doi:10.1186/s12870-022-03709-3)
Supplement: Supplementary file 5 — Additional file 5: Table S3. Primers for quantitative real-time PCR (qRT-PCR). [file 12870_2022_3709_MOESM5_ESM.docx]

**Table S3** Primers for quantitative real-time PCR (qRT-PCR)

| Gene name | Gene ID | Forward primer sequence (5’-3’) | Reverse primer sequence (5’-3’) |
| --- | --- | --- | --- |
| *CsTCP1* | Cs2g08080.1 | ACCCAGATGATGAGGAAGATG | TTATTAACGCCTAGAGCAACG |
| *CsTCP2* | Cs2g15820.1 | TCACGCAATCAGTTAGAGCC | TTGGGAGAAGAAGAGGAGGG |
| *CsTCP3* | Cs2g25640.1 | GGTGGCAATTTAGTAGGAAG | GTTGATGGACCAGAAGATGA |
| *CsTCP4* | Cs3g22260.1 | ACGGCTTCTGGATGCTACCG | GCTGATGACCGCCCATTTGA |
| *CsTCP5* | Cs5g03980.1 | GGTCAAGTATTAGTGGGAGTAGCG | CGGAAACCTGCCTGTATGGA |
| *CsTCP6* | Cs5g10130.1 | GCCCGTAGATTCTTACCAGG | ATTGTTTGCTGTGACCGTTG |
| *CsTCP7* | Cs5g12070.1 | AGGGCAAGGAAGTTGAAAGA | GGCTCCCAATGGTAGTAAGG |
| *CsTCP8* | Cs6g18940.1 | CGGCAGCCAATCATCAGACT | CCACTGCACTCGCATTGC |
| *CsTCP9* | Cs6g22270.1 | AGACGACCCTGTGCTACTTC | GGTCTTCTGTTTGGGATGTT |
| *CsTCP10* | Cs7g03980.1 | CTTCCAACTCCGATGGCTCT | GGCACCCGAAATGTTCCTAT |
| *CsTCP11* | Cs7g11120.1 | GGACTATTCCAGCCTCTATGC | GGTCCCAACCCTTCTATTTT |
| *CsTCP12* | Cs7g12770.1 | GGCTGAAGCAGAAGAGGAAG | TCTTGGCAGGTATCTGGGTT |
| *CsTCP13* | Cs7g25460.1 | CAGCCAGCAGTCCATCAATC | TGTTCCTCATCTTCGCCTTG |
| *CsTCP14* | Cs7g26250.1 | CTTCTTTATCATTCTTCCATTTC | AGGATCAGGTGTTAAGGGTT |
| *CsTCP15* | Cs8g16060.1 | CATGTCGCATTGCCGATAAC | AACTCTCTTCCGGCATTGTTTC |
| *CsTCP16* | Cs8g16080.1 | CAGAGTCACCATCATCAGCAAAG | CGTTCGTCGAGTTGCGTTT |
| *CsTCP17* | Cs9g12640.1 | CGGCAGCCAATCATCAGACT | CCACTGCACTCGCATTGC |
| *CsTCP18* | Cs9g16600.1 | TGGTGTCTTGGGTTCGTGTT | TCTGGGTAGTTGAAGTTGTGGTG |
| *CsTCP19* | orange1.1t02428.1 | TTCCCGCTGTGGCTGTATCC | GAACATCCCCTGCGACGAAA |
| *CsTCP20* | orange1.1t03896.1 | GCCACCTTCATCTTCCACAG | AACAGCACCTCCACTTCCTC |
| *CsActin* | Cs1g05000.1 | CCGACCGTATGAGCAAGGAAA | TTCCTGTGGACAATGGATGGA |
